# Modulation of Glutamate Transport and Receptor Binding by Glutamate Receptor Antagonists in EAE Rat Brain

**DOI:** 10.1371/journal.pone.0113954

**Published:** 2014-11-26

**Authors:** Grzegorz Sulkowski, Beata Dąbrowska-Bouta, Elżbieta Salińska, Lidia Strużyńska

**Affiliations:** 1 Laboratory of Pathoneurochemistry, Department of Neurochemistry, Mossakowski Medical Research Centre, Polish Academy of Sciences, Warsaw, Poland; 2 Laboratory of Pharmaconeurochemistry, Department of Neurochemistry, Mossakowski Medical Research Centre, Polish Academy of Sciences, Warsaw, Poland; Universidad de Castilla-La Mancha, Spain

## Abstract

The etiology of multiple sclerosis (MS) is currently unknown. However, one potential mechanism involved in the disease may be excitotoxicity. The elevation of glutamate in cerebrospinal fluid, as well as changes in the expression of glutamate receptors (iGluRs and mGluRs) and excitatory amino acid transporters (EAATs), have been observed in the brains of MS patients and animals subjected to experimental autoimmune encephalomyelitis (EAE), which is the predominant animal model used to investigate the pathophysiology of MS. In the present paper, the effects of glutamatergic receptor antagonists, including amantadine, memantine, LY 367583, and MPEP, on glutamate transport, the expression of mRNA of glutamate transporters (EAATs), the kinetic parameters of ligand binding to N-methyl-D-aspartate (NMDA) receptors, and the morphology of nerve endings in EAE rat brains were investigated. The extracellular level of glutamate in the brain is primarily regulated by astrocytic glutamate transporter 1 (GLT-1) and glutamate-aspartate transporter (GLAST). Excess glutamate is taken up from the synaptic space and metabolized by astrocytes. Thus, the extracellular level of glutamate decreases, which protects neurons from excitotoxicity. Our investigations showed changes in the expression of EAAT mRNA, glutamate transport (uptake and release) by synaptosomal and glial plasmalemmal vesicle fractions, and ligand binding to NMDA receptors; these effects were partially reversed after the treatment of EAE rats with the NMDA antagonists amantadine and memantine. The antagonists of group I metabotropic glutamate receptors (mGluRs), including LY 367385 and MPEP, did not exert any effect on the examined parameters. These results suggest that disturbances in these mechanisms may play a role in the processes associated with glutamate excitotoxicity and the progressive brain damage in EAE.

## Introduction

Multiple sclerosis (MS) is a chronic inflammatory and neurodegenerative disease of the CNS. The characteristic features of the disease include demyelinating areas in the white matter of the spinal cord and brain, which lead to disturbances in nerve transmission [1], [2]. The process of inflammation is accompanied by increased levels of soluble inflammatory cytokines and enhanced levels of glutamate and excitotoxicity. These mechanisms have also been proposed as major determinants of the neurodegeneration observed in MS and its animal model EAE [1], [3], [4], [5].

Enhanced levels of glutamate in the cerebrospinal fluid of MS patients and changes in the expression of ionotropic glutamate receptors (iGluRs) and metabotropic glutamate receptors (mGluRs) have been observed [6]. Furthermore, correlations between altered glutamate homeostasis, cell death, axonal damage, and disturbances in glutamatergic neurotransmission have been identified during MS/EAE pathology [7], [8], [9].

Axonal degeneration is an important problem during progressive neurological disability in MS/EAE. Glutamate kills neurons by excitotoxicity, which is caused by sustained activation of glutamate receptors and a subsequent massive influx of Ca^2+^ into viable neurons [10]. Calcium, which is the primary signaling agent involved in excitotoxic injury, may enter the cell via various mechanisms, but the most important mechanism is its entrance via ion channels coupled to NMDA receptors [11]. Other non-NMDA iGluRs (AMPA/kainate) and/or group I mGluRs may also be involved in glutamate-induced neuronal death [12], [13]. Recent studies have shown that glutamate can also be toxic to white matter oligodendrocytes and myelin via mechanisms triggered by these receptors activation [1], [2], [14]. The proper function of glutamate uptake is critical to prevent glutamate-induced brain cell damage, and drugs that regulate the function and expression of glutamate transporters (GluTs) and glutamate receptors (GluRs) may have a protective effect against excitotoxic cell death [2]. Thus, the strict regulation of extracellular glutamate levels appears to be one of the most promising therapeutic strategies to prevent neurodegeneration in MS/EAE [1], [15], [16], [17].

The level of extracellular glutamate in the brain must be strictly controlled, and this regulation is primarily accomplished by GluTs. Brain cells express a number of different proteins that transport glutamate. Some proteins are located on the extracellular plasma membrane, and some proteins are intracellular [18]. To date, five different “high-affinity” GluTs (GLT-1, GLAST, EAAC1, EAAT4, and EAAT5) have been cloned in rats and rabbits. All of these proteins provide Na^+^-K^+^-coupled transport of L-glutamate, as well as L- and D-aspartate. In the human brain, five homologous EAATs have been identified (EAAT1-EAAT5) [19], [20]. GLT-1 and GLAST are primarily expressed by astrocytes and oligodendrocytes; GLT-1 is highly expressed in the brain and is mainly responsible for glutamate uptake from the synaptic clefts in the forebrain and hippocampus. In the cerebellum, the glutamate level is regulated by GLAST [14], [21], [22]. Knockout studies with specific antisense oligonucleotides have demonstrated that the loss of GLT-1 produced excitotoxic neurodegeneration in the CNS [21]. In brain pathologies with neurodegenerative features, such as ALS (amyotrophic lateral sclerosis), MS, and traumatic brain injury, glial GLT-1 and GLAST are the primary determinants responsible for controlling the level of extracellular glutamate in the brain [23], [24], [25].

Previous *in vivo* and *in vitro* studies have provided evidence for the participation of glutamate excitotoxicity and the overstimulation of glutamate receptors (GluRs) in the pathophysiology of multiple chronic neurodegenerative disorders, such as ALS, Huntington's disease, Parkinson's disease, motor neuron disease (MND), MS/EAE, brain injury, and ischemia [10], [26]. These findings suggest that blockade of GluRs by their specific antagonists may exert a neuroprotective action. Many experiments have indicated that antagonists of NMDA receptors (e.g., amantadine and memantine) and antagonists of mGluRs G I (e.g., LY 367385 and MPEP) have a protective effect against excitotoxicity. Memantine has been shown to modify the neurological course of EAE and to prevent the breakdown of the blood brain barrier (BBB) [16]. The protection of cultured cerebellar granule neurons by the combined actions of NMDAR antagonists (amantadine and memantine) and mGluR G I antagonists (LY 367385 and MPEP) has also been observed [17].

In a previous study, we observed time-dependent changes in the protein expression of GluTs (GLT-1 and GLAST) in the forebrain and cerebellum of EAE rats [27]. We further investigated the effects of the GluR antagonists amantadine and memantine (noncompetitive NMDA receptor antagonists), as well as antagonists of group I mGluR LY 367385 (a competitive antagonist of mGluR1) and MPEP (a noncompetitive antagonist of mGluR5), on the development of neurological symptoms during EAE [28], [29]. The treatment of EAE rats with these antagonists modified the expression of mRNA and the protein levels of mGluR1, mGluR5, and NMDA receptors. The pharmacological inhibition of ionotropic NMDA receptors by amantadine and memantine, apart from the suppression of neurological symptoms in EAE rats, also reduced the expression of pro-inflammatory cytokines in the brain [29]. In contrast, the antagonists of group I mGluRs LY 367385 and MPEP did not affect the inflammatory process or the neurological condition of EAE rats [28], [29].

In the present study, we investigated whether amantadine and memantine and LY368573 and MPEP influenced the expression and function of GluTs in neuronal and glial fractions, as well as MK-801 binding to the membrane fraction in the acute phase of EAE. Ultrastructural observations of nerve endings during EAE and after treatment with GluR antagonists were conducted using transmission electron microscopy (TEM).

## Materials and Methods

### 1. Ethics Statement

This study was carried out in strict accordance with the regulations of the Experiments on Animals Act (Act of 21 January 2005 on experiments on live animals, the Parliament of the Republic of Poland, Dz. U. Nr 33, poz. 289); as well as with the Directive 2010/63/EU of the European Parliament and of the Council of the European Union of 22 September 2010 on the protection of animals used for scientific purposes. All animal experiments were approved by the Fourth Warsaw Local Ethics Committee for Animal Experimentation; permit number 61/2009. All surgery was performed under sodium pentobarbital anesthesia, and all efforts were made to minimize suffering.

### 2. Animal model

The experiments utilized female Lewis rats that weighed approximately 200 g. The rats were divided into 6 groups (control, EAE, and 4 EAE groups with different drug treatments: amantadine, memantine, LY 367385, or MPEP). To induce experimental autoimmune encephalomyelitis (EAE), we immunized the rats subcutaneously in both hind feet with an inoculum that contained guinea pig spinal cord homogenate emulsified in Freund's complete adjuvant containing 5.5 mg/mL *Mycobacterium tuberculosis* H37Ra (Difco, Detroit, MI, USA) as previously described [28], [29]. Amantadine (Sigma-Aldrich, Steinheim, Germany) and memantine (Sigma-Aldrich, Steinheim, Germany) were administered at a dose of 100 mg/kg body weight (b.w.)/day and 60 mg/kg b.w./day, respectively. Both LY 367385 (Tocris, Bristol, UK) and MPEP (Tocris, Bristol, UK) were administered at a dose of 10 mg/kg b.w./day. The drugs were administered via an intraperitoneal injection to the EAE rats according to the previously described procedure [28], [29].

Rats were housed under environmentally controlled conditions and had unrestricted access to food and water. Body weights and neurological deficits were measured daily according to the following scale: 0 = no signs, 1 = flaccid tail, 2 = impairment of fighting reflex and/or loss of muscle tone in hind limbs, 3 = complete paralysis of hind limbs, 4 = paraplegia, and 5 = moribund state/death [30], [31]. Sham-immunized rats (control group) received subcutaneous injections of Freund's complete adjuvant that contained only *M. tuberculosis* (Difco, Detroit, MI, USA). All experiments were performed in the acute phase of the disease (at 12 d.p.i.).

### 3. Materials

During the experiments, the rats were monitored and weighed daily after the initial immunizing injection or after drug administration between 5 and 11 d.p.i. At 12 d.p.i., four rats from each group were killed to obtain tissue for real-time PCR analyses, and an additional four rats per group were used for the preparation of membrane fractions. The brains were rapidly removed, and the tissues were then frozen in liquid nitrogen and stored at −70°C for further experiments. Brain fractions were prepared from fresh tissue.

### 4. Preparation of synaptosomal fraction

Synaptosomes were isolated according to the method of Booth and Clark [32] with centrifugation in a discontinuous Ficoll gradient (7%, 12%) at 99,000 g. The synaptosomes obtained by this procedure were highly pure and had well-maintained energy metabolism; therefore, they are considered to be a good model for nerve endings [32]. Fractions were used for [^3^H] glutamate transport (uptake and release) measurements.

### 5. Preparation of glial fraction

Glial plasmalemmal vesicle (GPV) fractions were isolated according to the method of Daniels and Vickroy [33] as described and characterized in our previous papers [34], [35]. Briefly, the brains were homogenized in 30 ml of isolation medium (0.32 M sucrose and 1 mM EDTA) and centrifuged at 1,000 g for 10 min. The supernatant was diluted using SEDH medium (0.32 M sucrose, 1 mM EDTA, 0.25 mM dithiothreitol, and 20 mM HEPES, pH 7.4) and centrifuged at 5,000 g for 15 min. After several additional fractionations, the material was centrifuged in a three-step discontinuous Percoll gradient (20%: 10%: 6%) for 10 min at 33,500 g. The layer between 0% and 6% Percoll was collected to obtain the GPV used for further examination of [^3^H] glutamate transport.

### 6. [^3^H] glutamate transport assay

The protein concentration was determined by the method of Lowry [36]. Synaptosomal and GPV fractions were used to measure Na^+^-dependent [^3^H] glutamate uptake and KCl-dependent release of accumulated amino acids. Radioactive glutamate accumulation was performed according to the filtration method described by Divac [37]. Radioactivity trapped on the filters was then measured in a liquid scintillation counter (Wallack 1409). In the case of release, 50 mM KCl was used at a maximum of the uptake curves (4 min), and liberated radioactivity was assayed after 6 min. To prevent the conversion of glutamate to α-ketoglutarate, aminooxyacetic acid (AOAA), which is an inhibitor of AAT (aspartate aminotransferase), was added [38].

### 7. Determination of the mRNA levels of EAATs by real-time PCR

Total RNA was extracted from the brain cortex of control and EAE rats according to the method of Chomczyński and Sacchi [39]. Isolation was performed using TRI Reagent (Sigma, St. Louis, MO, USA). Reverse transcription of 2 µg of total RNA was performed in a final volume of 20 µL using random primers and avian myeloblastosis virus (AMV) reverse transcriptase (Applied Biosystems, Forest City, CA, USA). The RT-PCR conditions were as follows: reverse transcription at 42°C for 45 min and denaturation at 94°C for 30 s. For quantitative real-time PCR analysis, TaqMan technology was applied. The rat GluT-specific primers used were as follows: for GLAST (EAAT1), ID: Rn00570130_m1*, gen symbol Slc1a3; for GLT-1 (EAAT2), ID: Rn00691548_m1, gen symbol Slc1a2*; and for EAAC1 (EAAT3), ID: Rn 00564705_m1*, gen symbol Slc1a1. The probes were obtained from Applied Biosystems (Forest City, CA, USA). The mRNA expression levels of GluTs and actin were determined using the pre-validated TaqMan assay reagents (Applied Biosystems, Forest City, CA, USA). Real-time PCR was conducted on an ABI Prism 7500 system using 5 µL of RT product, TaqMan PCR Master Mix, primers, and a TaqMan probe in a total volume of 20 µL. The PCR cycle conditions were as follows: initial denaturation at 95°C for 10 min, 50 cycles of 95°C for 15 s, and 60°C for 1 min. Each sample was analyzed in triplicate. The relative expression levels of the GluT mRNAs were calculated using the standard curve method and normalized to actin.

### 8. Membrane preparation and [^3^H] MK-801 binding assay

A crude cortical membrane fraction that contained NMDA receptors was isolated from the cerebral cortices with hippocamp from Lewis rat brains as previously described by Wang [40]. Prior to each experiment, the frozen pellets were thawed and washed twice in Tris-HEPES buffer that contained EDTA and twice in Tris-HEPES buffer without EDTA to remove endogenous amino acids. The assay tubes contained membranes (200 µg of protein per tube), 4 nM [^3^H] MK-801, 10 µM NMDA, 10 µM glycine, and different concentrations of amantadine (0–2000 µM) and memantine (0–1000 µM). The samples were incubated at 28°C for 1 h, and the incubation was terminated by rapid filtration on Whatman GF/B filters (Whatman International Ltd, Maidstone, England) using a Brandel M-24 cell Harvester (Labequip, Ontario, Canada). Radioactivity was measured by liquid scintillation spectrometry using a Wallac 1409 Counter. Non-specific binding was determined in the presence of 10 µM unlabeled MK-801. The assays were performed in triplicate. The data analyses were individually performed for each rat using the computer program PRISM from GraphPad (GraphPad Software, La Jolla, USA).

### 9. Electron microscopic studies

The estimation of morphological changes in the brain was performed at 12 d.p.i. using rats (n = 5) from each experimental group (control, EAE, EAE+amantadine, EAE+memantine, EAE+LY 368573, and EAE+MPEP). The animals were anaesthetized and perfused through the heart with fixative solution (2% paraformaldehyde, 2.5% glutaraldehyde, and 0.1 M cacodylate buffer, pH 7.4). After perfusion, small specimens from the forebrain were fixed overnight in the same solution and then fixed in 1.5% OsO_4_ and 0.8% K_4_(FeCN)_6_ for 2 h. After dehydration in ethanol and propylene oxide, the sample was subsequently embedded in Spurr resin, and ultrathin sections were examined using a JEM 1200 Ex electron microscope.

### 10. Statistical analysis

The results are expressed as the means ± SD from 3–4 experiments. Significance was assessed by one-way-ANOVA. Dunnett's multiple comparison test was used to identify the changes that were significantly different compared with the control or EAE values (*P<0.05, **P<0.01, ***P<0.001 vs. control rats; ^#^P<0.05, ^##^P<0.01, ^###^P<0.001 vs. EAE animals).

## Results

### 1. The influence of drugs on the course of EAE

We identified changes in body weight in the drug-treated and untreated EAE rats. Rats in all experimental groups underwent a progressive 20–40% weight loss compared with the control animals (data not shown). A statistically significant increase in body weight (about 15%) compared to EAE animals was observed in rats treated with amantadine and memantine (Table 1).

**Table 1:** Characterization of the EAE animal model and the clinical parameters of EAE rats prior to and after treatment with antagonists of glutamatergic receptors.

	EAE	Amantadine	Memantine	LY 367385	MPEP
Animals with clinical symptoms (%)	95.66	98.75	97.50	95.94	98.47
Animals with severe EAE (%)	74.20	60.70*	62.80*	73.90	71.30
Lethality (%)	4.00	1.25	2.50	3.07	1.53
Inductive phase (days)	10.6±2.3	12.0±1.2*	12.1±1.9*	10.7±1.3	10.6±1.4
Body weight at 12 d.p.i. (g)	141.5±0.5	165.5±1.1*	161.3±3.4*	141.7±4.1	136.5±4.5
Maximal CI (score)	4.5±0.2	2.3±0.3*	2.5±0.4*	4.1±0.7	4.0±0.6
Cumulative CI (score)	28.4±4.8	19.5±1.7*	20.2±2.3*	26.9±0.4	27.5±0.8
Duration of disease (days)	21.3±1.6	17.8±1.4*	16.7±2.1*	20.6±1.6	21.1±1.3
Number of animals	225	80	80	65	65

The values represent the means ± SD. *P<0.05 indicates significant differences compared with the EAE rats. Combined administration of LY 367385 or MPEP in combination with the NMDAR antagonists (amantadine and memantine) did not influence the neurological deficits or the condition of the experimental rats during the course of the disease. The neurological deficits and condition of the examined animals were the same as in the case of treatment with amantadine or memantine exclusively (data not presented). CI – cumulative index.

After the administration of both amantadine and memantine, we observed a reduction in the severity and duration of the neurological deficits. All rats in these two experimental groups exhibited a better physiological condition compared with the EAE animals. We noticed a reduction in the severity and duration of neurological deficits. The maximal disease score was reduced to 2+ (whereas in EAE rats, it was 4.5+). The average cumulative index, duration of illness, and maximal score were reduced by factors of 8.5, 4.0, and 2.1, respectively, relative to those of the EAE rats. The duration of the acute phase of the disease was also reduced by 1-2 days compared with that of the untreated EAE rats (Table 1). We did not observe neuroprotective effects of LY 367385 or MPEP on the neurological deficits, the condition of the experimental animals, or the duration of the disease. The changes in lethality observed in rats treated with MPEP were not statistically significant.

Detailed observations of the EAE animals and the clinical parameters during the experiment, as well as the effects of GluR antagonist administration on neurological deficits during the course of EAE, are presented in Table 1. The effects are also described and illustrated in detail in our previous publications [28], [29].

### 2. Glutamate transport

**Figure 1:**
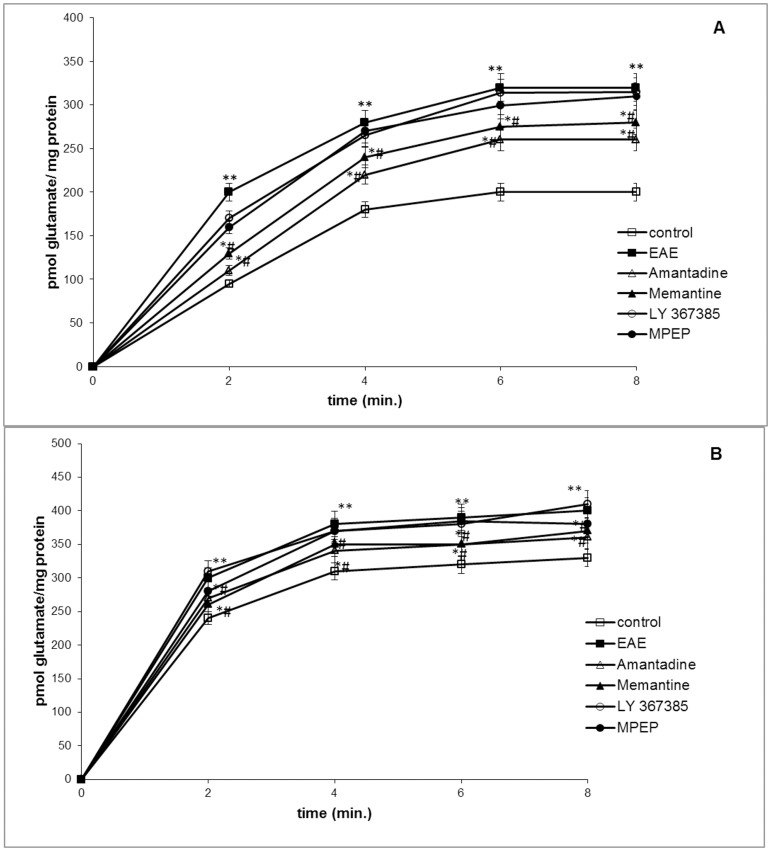
Na+-dependent [^3^H] glutamate uptake into synaptosomes (A) and the GPV fraction (B) obtained from the control, EAE, EAE+amantadine, EAE+memantine, EAE+LY367385, and EAE+MPEP rat brains during the acute phase of the disease (12 d.p.i.). Results represent the mean values ± SD from five separate experiments performed in duplicate; * P<0.05, ** P<0.01, significantly different compared with the control group; # P<0.05 vs. EAE rats not subjected to therapy (Student's t-test).

**Figure 2:**
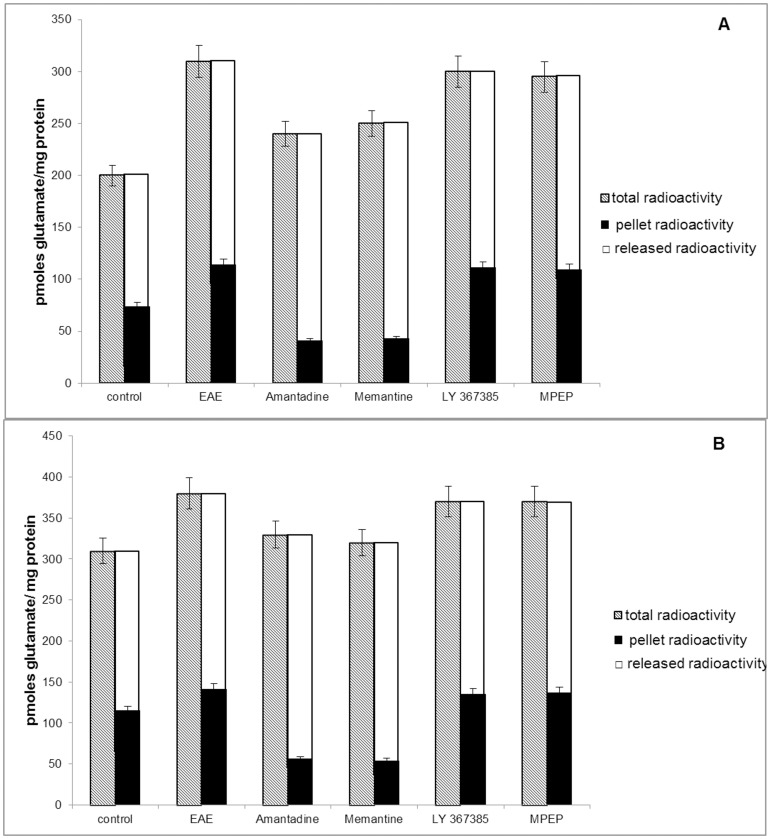
Glutamate release from synaptosomes (A) and GPV fraction (B) obtained from control, EAE, EAE+amantadine, EAE+memantine, EAE+LY367385, and EAE+MPEP rat brains during the acute phase of the disease (12 d.p.i.). Bars present total radioactivity in pellet (KCl no added), radioactivity remaining in pellet after depolarization by KCl (pellet radioactivity), and released of [^3^H] glutamate radioactivity from pellet after depolarization by KCl. Membranes were depolarized with 50 mM KCl at a maximum of the uptake curves (6 min), and radioactivity was assayed after 6 min. Results represent the means ± SD from five separate experiments performed in triplicate; * P<0.05 significantly different from the spontaneous release control group; # P<0.05 vs. EAE rats not subjected to therapy (Student's t-test).

The kinetic and pharmacological properties of sodium-dependent [^3^H] glutamate transport (uptake and release) in synaptosomal and GPV fractions were analyzed at the peak of the disease at 12 d.p.i. The rate of radioactive glutamate uptake into synaptosomal and GPV fractions was significantly enhanced in the EAE rats compared with the controls by approximately 60% (Fig. 1A) and 20% (Fig. 1B), respectively. Treatment with amantadine and memantine decreased glutamate uptake in the synaptosomes by approximately 20% relative to the EAE rats, but the level of accumulated glutamate was higher (30–40%) relatively to that of the control rats (Fig. 1A). A similar trend was observed for the GPV fraction (Fig. 1B). The stimulated release of glutamate changed within a similar range in both fractions compared with the respective control values. After amantadine and memantine treatment, we observed an increase in the release of previously accumulated [^3^H] glutamate from the synaptosomal fraction by approximately 30% (Fig. 2A), whereas in the GPV fraction, it rose by approximately 20% (Fig. 2B) compared with the respective controls. Treatment of EAE rats with mGluR G I antagonists (LY 367385 and MPEP) did not display a noticeable effect on glutamate transport (uptake or release) in synaptosomal or GPV fractions (Fig. 1A, 1B, 2A, 2B).

### 3. Inhibition of [^3^H]MK-801 binding by glutamate receptor antagonists

**Figure 3:**
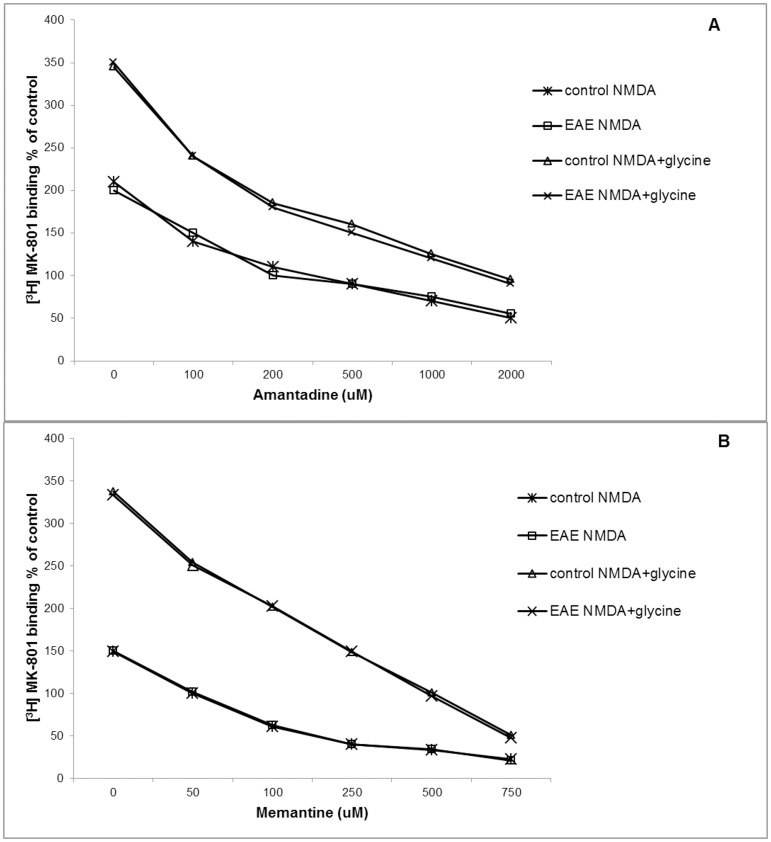
The inhibition of [^3^H]MK-801 binding to rat brain membranes by amantadine (A) and memantine (B). Labeled [^3^H]MK-801 was used at 4 nM; NMDA was used at 10 µM either without or with glycine at 10 µM. The specific binding was determined in the presence of 10 µM unlabeled MK-801. Results are expressed as a percentage of the control (mean ± SD) from three separate experiments performed in triplicate; * P<0.05 significantly different from the control group (Student's t-test).

We did not identify differences in the kinetic parameters of MK-801 binding to the membrane fractions obtained from the control and EAE rats (Fig. 3A, B). Both tested NMDA receptor antagonists (amantadine and memantine) inhibited [^3^H]MK-801 binding to the rat brain membranes (obtained from control and EAE rats) in a concentration-dependent manner (50-1000 µM). Both compounds exerted an inhibitory effect in the absence and in the presence of glycine, which effectively increased the MK-801 binding (Fig. 3A, B). As it was expected antagonists of group I mGluR (LY 367385 and MPEP) did not modify MK-801 binding to the rat brain membranes (data not presented).

### 4. Changes in the expression of glutamate transporters

Real-time PCR analysis was used to investigate the changes in mRNA levels of the GluTs during the course of EAE and after treatment with GluR antagonists.

**Figure 4:**
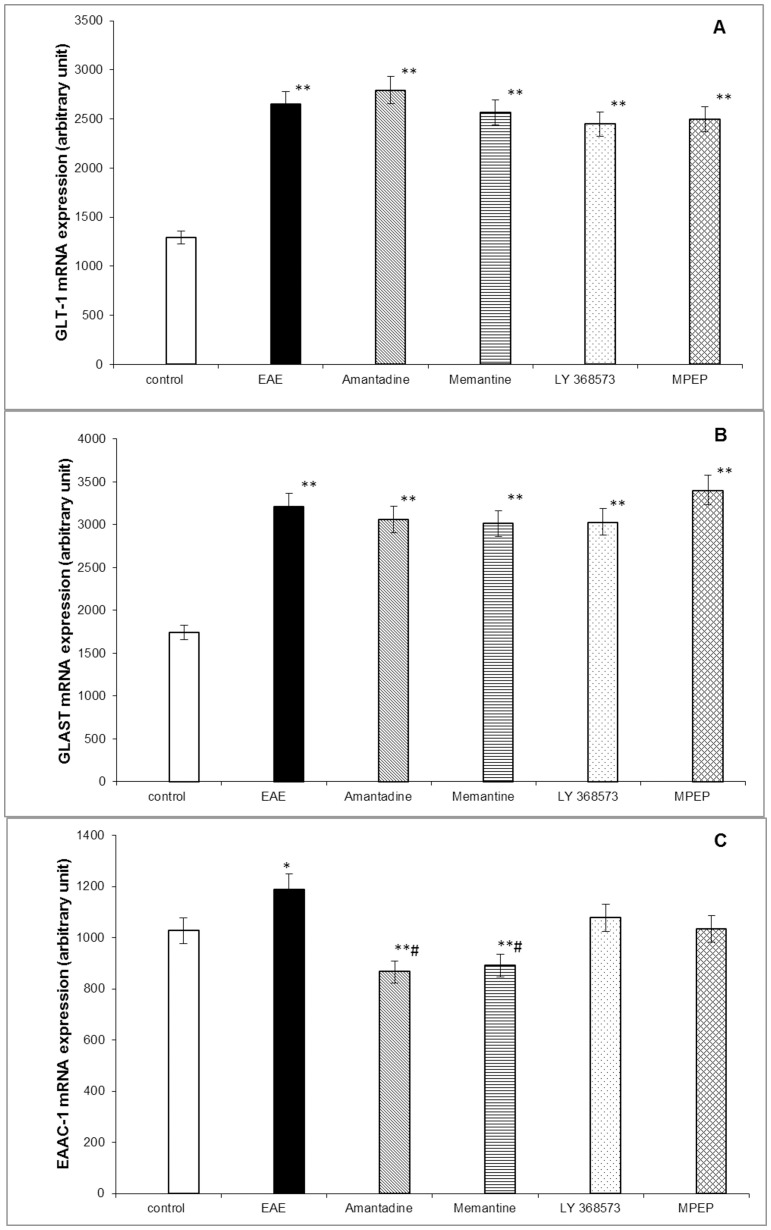
Expression of glutamate transporter (GluT) mRNA in the forebrains of the control, EAE, and EAE rats after treatment with antagonists of the glutamatergic receptors (amantadine, memantine, LY 367385, and MPEP) in the acute phase of EAE. The mRNA levels (A)-GLT-1; (B)-GLAST; (C)-EAAC-1 were determined by quantitative real-time PCR (see Materials and Methods) and normalized to actin. Graphs indicate the results expressed as a percentage of the control from four independent experiments. The values represent the means ± SD. * P<0.05, ** P<0.01, significantly different compared with the control rats. # P<0.05, ## p<0.01 compared with the EAE rats not subjected to therapy (one-way ANOVA followed by Dunnett's multiple comparison post-hoc test).

We analyzed the mRNA level of three main excitatory amino acid transporters (EAATs) expressed in the rat brain, glial (GLT-1, GLAST) and neuronal (EAAC-1), to identified changes in the immunized rats. At the peak of the disease (12 d.p.i.), we observed a significant increase in GLT-1 and GLAST mRNA, which reached about 200% of the control value (Fig. 4A and 4B). In contrast, the expression of EAAC-1 was approximately 15% higher relative to the control level (Fig. 4C).

After the administration of amantadine or memantine, the animals that developed EAE exhibited lower EAAC-1 mRNA levels (by approximately 20% compared with the control and by approximately 30% compared with the untreated EAE rats (Fig. 4C)). The expression of GLT-1 and GLAST mRNA was practically unchanged compared with their expression in the EAE rats after treatment with amantadine or memantine (Fig. 4A and Fig. 4B). After the application of amantadine or memantine, the level of EAAC-1 mRNA decreased by approximately 25–30% compared with that in the EAE rats (Fig. 4C), and was not significantly different compared with the control level.

### 5. Electron microscopy

**Figure 5:**
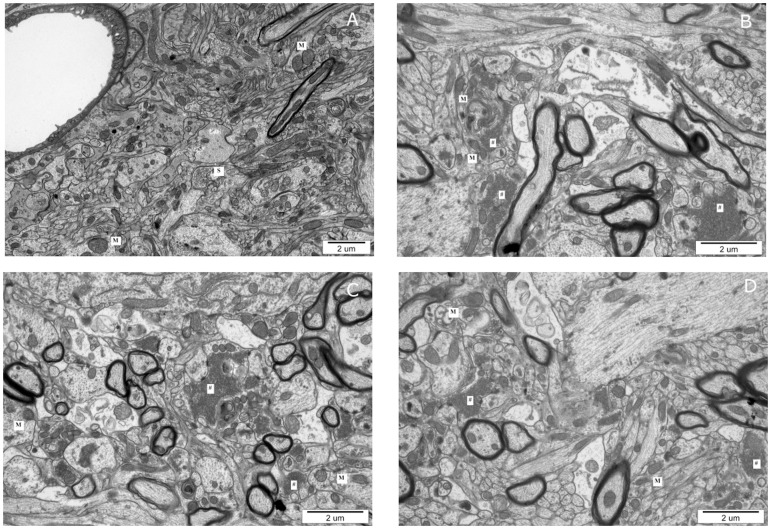
Representative electron micrographs of synapses in the forebrain specimens obtained from the control rats (A), EAE rats at 12 d.p.i. (B), EAE+amantadine-treated rats at 12 d.p.i. (C), and EAE+LY 367385-treated rats at 12 d.p.i. (D). M - mitochondria, S - synaptic vesicles, # - synaptic vesicles accumulation in the extra-synaptic space.

The electron microscopy studies were performed in forebrain specimens obtained from rats during the acute phase of EAE (at 12 d.p.i.). In these studies, we evaluated the appearance of the nerve endings. In the brains of the control rats, we did not observe abnormalities associated with the synapses, which showed a normal mitochondrial morphology and a typical number of synaptic vesicles (Fig. 5A).

In the brains of animals assessed during the acute phase of disease, we observed signs of synaptic degeneration and abnormalities. Synaptic mitochondria exhibited an abnormal structure, i.e., loss of the internal mitochondrial membrane integrity and a lower density of the mitochondrial matrix (Fig. 5B). We observed synaptic vesicle accumulation in the extra-synaptic space as a result of synaptic membrane disintegration (Fig. 5B). The administration of NMDAR antagonists (amantadine and memantine) and mGluR G I (LY 367385 and MPEP) did not improve the morphology of synapses during the acute phase of EAE (Fig. 5 C and D). Ultrastructural images of the brains after treatment with tested antagonists were similar to those obtained from EAE rats.

## Discussion

Pharmacological investigations strongly suggest that NMDA and mGluRs G I are involved in the pathogenesis of EAE. The administration of MK-801 (an antagonist of NMDA receptors) improved the neurological status of EAE rats [41], but clinical use of MK-801 has been limited because of its side effects. Aminoadamantances (amantadine and memantine) are NMDAR antagonists that are structurally distinct from MK-801 and have been found to be better tolerated by experimental animals than MK-801. In addition, both drugs have been used as treatments for dementia and Parkinson's disease with good tolerance [42]. Thus, we utilized the NMDAR antagonists amantadine and its derivative memantine, as well as the mGluRs G I antagonists LY 367385 and MPEP, for the development of new neuroprotective strategies that can be used to treat MS/EAE.

The current study also demonstrated changes in glutamate transport and the expression of mRNA for specific GluTs, alterations in MK-801 ligand binding to specific NMDA receptors, and ultrastructural disturbances in nerve endings during the clinical course of EAE. We analyzed the potential therapeutic effects of the GluR antagonists *in vivo*, and we demonstrated that amantadine and memantine effectively reduced the development of neurological deficits and the duration of the disease. Both substances had similar effects on all tested parameters that described the state of the animals and characterized the disease. The maximum score (CI) of the disease decreased to 2.3 in amantadine-treated rats and to 2.5 in memantine-treated rats, but in the untreated EAE animals, the score remained at 4.5. Other parameters were also changed after therapy. The duration of the disease was reduced by approximately 2–3 days, whereas the inductive phase was prolonged by 2 days relative to the EAE rats.

The neuroprotection of NMDAR antagonists during excitotoxic neuron injury is most likely related to the blockade of calcium influx into the cells via the receptor's channels. The current experiments confirmed the dose-dependent inhibitory activity of amantadine and memantine on MK-801 binding to the membrane fraction isolated both from control and EAE animals. Treatment with antagonists of the group I mGluRs did not exert visible effects on the physiological conditions or other tested parameters of the EAE rats.

The electron microscopy studies demonstrated the degeneration of synapses. In the acute phase of EAE, we observed an accumulation of synaptic vesicles in the neuropil that was outside the disintegrated synaptic membranes. Treatment with both groups of glutamatergic receptor antagonists did not improve the condition of the nerve endings, and the degenerative process remained prominent. A large number of synaptic vesicles that accumulated outside the synaptic space were observed after the administration of NMDAR antagonists. These morphological changes confirmed the disturbances in synaptic transport detected at the biochemical level.

Previously published findings, including our own results, have suggested that both subtypes of glutamatergic receptors (ionotropic and metabotropic) could be involved and cooperate in the excitotoxic damage of the different models of excitotoxicity and during the pathology of EAE [28], [29], [43], [44]. The results reported in the present work indicate that the expression of mRNA for the tested GLT-1, GLAST, and EAAC1 increased in the forebrain of the EAE rats during the acute phase of the disease (12 d.p.i.). The levels of mRNA for GLT-1 and GLAST (EAATs primarily expressed on glial cells) increased 2-fold compared with the respective control. Our results are in accordance with the findings reported by Ought who also observed increase of EAATs mRNA during acute phase of EAE [30]. Moreover, our data indicate a correlation between the enhancement of mRNA levels for the EAATs and increased glutamate uptake by the synaptosomal and GPV fractions. This up-regulation in GluT mRNA levels suggests that these alterations are a secondary response to the pathological changes at the glutamate level during the very early stages of EAE [27], [45]. However, the release of glutamate from both tested fractions was also enhanced. This finding may suggest the impairment of glutamatergic transmission, which can lead to the elevation of extracellular glutamate during EAE. The enhancement of glutamate uptake and the overexpression of mRNA for GluTs are most likely compensatory mechanisms against the increased glutamate levels during the course of EAE. After treatment with amantadine and memantine, the GluT returned to control conditions.

The observed neuroprotective effects of glutamate antagonists (amantadine and memantine) were most likely caused by the inhibition of NMDA receptors. Thus, we performed a dose-dependent assay of [^3^H]MK-801 binding to the rat brain membrane fractions in the *in vitro* experiments. Our results confirmed that both tested substances (amantadine and memantine) directly inhibited the activity of NMDA receptors and modulated the activity of NMDA channels. This observation is in accordance with ours early published data where we noticed unchanged level of protein and mRNA of NMDARs at acute phase of EAE [28]. The presence of glycine effectively increased the MK-801 binding to the membrane fractions. The site of MK-801 binding in the NMDA receptor complex in membranes is located inside the channel [46], [47]. Our experiments confirmed that the presence of glutamate and glycine is necessary for the maximal activation of NMDARs. The neuroprotective mechanisms of amantadine and memantine on the activity of NMDA receptors during EAE pathology are not completely understood and require further investigation.

## Conclusions

In conclusion, our findings confirm the involvement of EAATs as the compensatory mechanism operating against excitotoxic brain injury during the acute phase of EAE. We observed the overexpression of GLT-1, GLAST, and EAAC1 mRNA levels and the activity of transporters (i.e., glutamate uptake by neuronal and astroglial fractions was enhanced at 12 d.p.i. with a simultaneous increase in the release of this neurotransmitter).

Our studies demonstrated that the treatment of EAE rats with amantadine and memantine, but not with antagonists of group I mGluRs (LY 367385 and MPEP), had protective effects on the neurological deficits and improved the physiological condition of the immunized animals. Treatment with amantadine and memantine modulated glutamate transport, thereby decreasing glutamate uptake and release and reducing the mRNA levels of the EAAC-1 transporter, but did not affect the mRNA levels of the GLT-1 and GLAST transporters. Aminoadamantaces also had a dose-dependent effect on the modulation of MK-801 binding to NMDA receptors. However, the electron microscopy studies revealed the degeneration of nerve endings in the brains of EAE rats that did not improve after therapy with GluR antagonists. Thus, current therapies that suppress inflammation or glutamate excitotoxicity are partially effective when administered at an early stage of EAE (the asymptomatic phase of the disease).
